# A failed attempt at developing a search filter for systematic review methodology articles in Ovid Embase

**DOI:** 10.5195/jmla.2019.519

**Published:** 2019-04-01

**Authors:** Christine Neilson, Mê-Linh Lê

**Affiliations:** Neil John Maclean Health Sciences Library, University of Manitoba–Winnipeg, Canada, christine.neilson@umanitoba.ca; Neil John Maclean Health Sciences Library, University of Manitoba–Winnipeg, Canada, me-linh.le@umanitoba.ca

## Abstract

**Objectives:**

This paper describes the development, execution, and subsequent failure of an attempt to create an Ovid Embase search filter for locating systematic review methodology articles.

**Methods:**

The authors devised a work plan, based on best practices, for search filter development that has been outlined in the literature. Three reference samples were gathered by identifying the OVID Embase records for specific articles that were included in the PubMed Systematic Review Methods subset. The first sample was analyzed to develop a set of keywords and subject headings to include in the search filter. The second and third samples would have been used to calibrate the search filter and to calculate filter sensitivity and precision, respectively.

**Results:**

Technical shortcomings, database indexing practices, and the fuzzy nature of keyword terminology relevant to the topic prevented us from designing the search filter.

**Conclusion:**

Creating a search filter to identify systematic review methodology articles in Ovid Embase is not possible at this time.

## INTRODUCTION

The amount of health research literature published every year grows rapidly. Embase alone adds an average of 6,000 records per working day [1]. This volume of material presents challenges for locating articles related to and staying informed about a specific topic area. Search filters, or hedges, are established search strings that can be incorporated into a literature search to focus the results in a specific way. They can be found published in journal articles and on the websites of research and knowledge synthesis organizations like the McMaster University Health Information Research Unit [2], Scottish Intercollegiate Guidelines Network [3], and InterTASC Information Specialists’ Sub-Group [4].

Search filters that target articles about studies utilizing a particular methodology (e.g., randomized controlled trials, observational studies) or in a specific subject area (e.g., geographic location, cultural or ethnographic background, patient population) are currently available. However, there are no published filters for articles that deal with research methodology as the topic of the article itself. A filter of this nature would be beneficial to a variety of users—including researchers, methodologists, information professionals, or students—who are interested in systematic review methodology as a field of study or are directly involved in completing a systematic review and desire methodological guidance.

An alternative finding aid for locating systematic review methodology articles in PubMed was released in December 2015. The Systematic Review Methods subset for PubMed (sysrev_methods (sb)) was a curated list generated by the Scientific Resource Center that could be used to limit search results rather than a set of search terms that could be applied as a filter [5]. This subset focused on research methodology for systematic reviews and study types that were incorporated into systematic reviews, including comparative evaluations of techniques; development, evaluation, or validation of a technique; analyses of methods used by studies; and consensus and delphi studies and surveys of methods.

Although the Systematic Review Methods subset for PubMed was available at the time when the present project began, there was no equivalent tool available for Embase, a resource recommended as a complement to PubMed/MEDLINE searches, in part due to its broader international coverage of research literature [6]. The authors attempted to address this gap by designing a filter for locating systematic review methodology articles in Ovid Embase. Whereas the PubMed subset was populated using a variety of methods, including human screening and alerts (Paytner, pers. comm), we thought it would be possible to use a traditional search filter to streamline this process for Embase and enable all users quick access to methodological articles. Here, we describe our experience attempting to design a filter for isolating methodology articles in Ovid Embase following the four stages of filter development laid out by Jenkins: reference sample collection, search term selection, search filter evaluation, and search filter validation [7].

## METHODS

Prior to the start of our project, we conducted a review of the literature to determine best practices for search filter development. We also communicated with the Agency for Healthcare Research and Quality Scientific Resource Center Methods Library, which maintained the PubMed subset, about our proposed project, the creation and maintenance of their subset, and potential search terms (Paytner, pers. comm). Based on the information that we obtained, we created a four-stage work plan.

### Stage one: Reference sample collection (completed)

We collected citations for articles that were independently identified as methodologically focused from PubMed using the PubMed Systematic Review Methods subset in the summer of 2017. The corresponding Embase records were identified for inclusion in our reference samples, where possible, through PubMed identifier (PMID) and title searches in Embase (Ovid). Because good practice is to develop a search filter with one reference sample and then validate that filter with another reference sample of different records [8, 9], we divided the citations into three separate reference samples:

1. a development sample for identifying a set of keywords and subject headings for inclusion in the search filter; development sample years were 2001, 2011, and 2016
2. a calibration sample for examining records that were not retrieved by the filter to determine the reasons for exclusion, allowing the search filter to be modified as appropriate; calibration sample years were 2000, 2010, and 2015
3. a validation sample to calculate the precision and sensitivity of the completed search filter; validation sample years were 1999, 2009, and 2014

We chose to limit citations that were included in the reference samples to clusters of publication years at five-year intervals to reflect potential terminology changes over time. That is, each reference sample contained records that were from a recent year (2016, 2015, or 2014), records from publications that were five years older (2011, 2010 or 2009), and records from publications that were ten years older (2001, 2000, or 1999). The sample sizes for the reference samples are shown in Table 1.

**Table 1:**
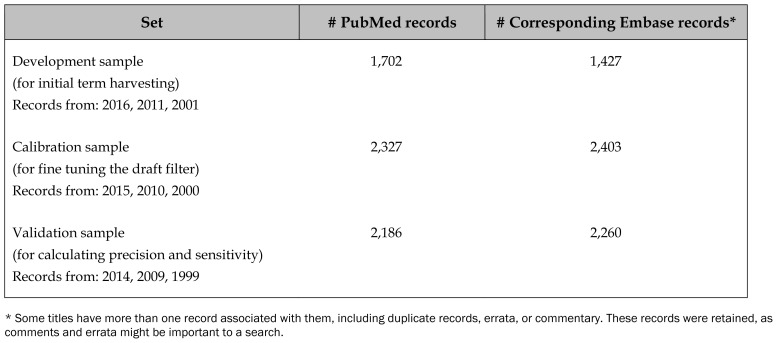
Sample sizes for the three reference samples collected based on identified PubMed records

Set	# PubMed records	# Corresponding Embase records*
Development sample (for initial term harvesting) Records from: 2016, 2011, 2001	1,702	1,427
Calibration sample (for fine tuning the draft filter) Records from: 2015, 2010, 2000	2,327	2,403
Validation sample (for calculating precision and sensitivity) Records from: 2014, 2009, 1999	2,186	2,260

* Some titles have more than one record associated with them, including duplicate records, errata, or commentary. These records were retained, as comments and errata might be important to a search.

### Stage two: Search term selection (initiated, not completed)

We used a combination of manual (subjective) and automated (objective) processes to identify relevant Emtree subject headings and keywords in the development sample. We used VOSviewer software to visualize the relationships of common terms. Sample searches combining individual potential terms with terms related to knowledge synthesis (e.g., systematic review, scoping review, rapid review, meta-analyses, and guidelines) were planned to compare the number of results that were focused on research methods as a topic versus those that were obtained in error, which would inform selection of terms for the search filter.

### Stage three: Search filter evaluation (not completed)

We planned to develop the Embase search filter through iterative testing, with the goal of developing a filter that retrieved the most previously identified citations for the sample years we examined, while retrieving a minimal number of extraneous results.

### Stage four: Search filter validation (not completed)

Once the search filter was finalized, we planned to validate the filter by running it in Embase, limiting the results to the years used for the validation sample, and comparing the search results to the validation sample. Calculations of sensitivity (the percentage of sample records retrieved in the search results) and precision (the percentage of relevant records retrieved in the results overall) would have followed. Precision calculations require manual review of citations to determine the relevance of individual citations retrieved. We would have determined the relevance of citations retrieved independently, with disagreements resolved through consensus. Our decisions about article relevance would have been based on the information presented in the article records and, where necessary, the full text of the article in question.

## RESULTS

### Search term development

Using the data visualization tool VOSViewer, a frequency analysis for commonly used keyword terms and subject headings (both phrase and single terms) in the 1,427 records that made up the development sample revealed 9 keywords and phrases that occurred in the subject fields of ≥15 articles, 82 keywords and phrases that occurred in the title of ≥15 articles, and 462 keywords and phrases that occurred in the abstract of ≥15 articles. VOSViewer does not double-count individual terms and phrases because it recognizes when terms appear together as a phrase and counts that as a single entity, which means that the number of times “review” and “systematic review” occur do not necessarily add up. The top 20 terms as identified by VOSViewer for the subject heading, title, and abstract fields are listed in Tables 1–3 in the supplemental appendix.

**Figure 1:**
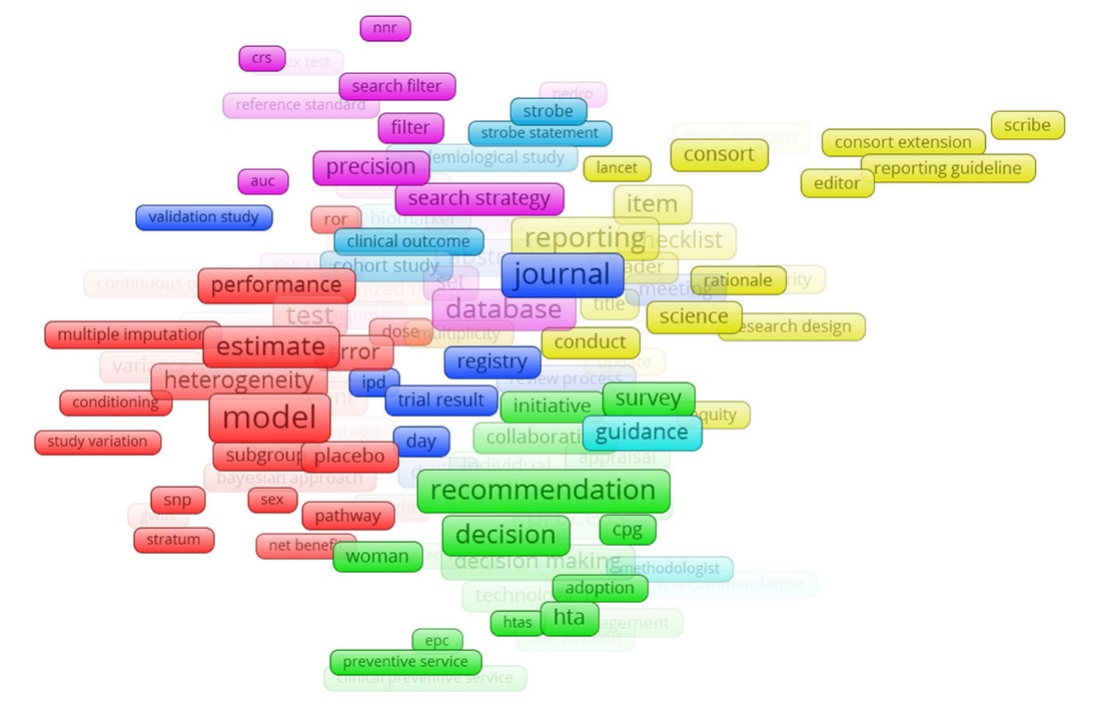
Frequency and relationships of terms commonly included in development sample abstracts, as depicted in VOSViewer

Visual representation of the frequency analysis (Figure 1) suggested that some terms were often associated with one another. For example, the purple group of terms—including “search filter,” “search strategy,” “database,” and “precision”—corresponded to terms dealing with literature searching and, potentially, search filter development. Other groups of terms, however—such as the dark blue terms “validation study,” “journal,” “trial result,” “day,” and “ipd”—were less suggestive of a coherent theme.

After potential filter terms were selected, we began testing them in Embase by combining them with common terms found in knowledge synthesis papers, such as “network meta analysis,” “publication standards,” “publication bias,” “rapid review,” and “guidelines” using the Boolean AND. During these tests, we noticed that database indexing did not behave as expected. For example, although the Embase subject heading “systematic review (topic)/” includes the following scope note indicating that it should be “used for items that discuss systematic reviews, but which are not themselves systematic reviews,” we found this subject heading had been applied to actual systematic reviews. For example, the article “The Experience of Caregivers Living with Cancer Patients: A Systematic Review and Meta-Synthesis” [10] is a systematic review of the literature rather than a methods piece, but “systematic review (topic)/” and “meta analysis (topic)/” were listed among its subject headings.

### Technical obstacles to filter development

Technical obstacles hindered our project. When compiling our reference samples, we initially ran into difficulty identifying some records via PMID in the Ovid Embase PM field. In some cases, a subsequent title search retrieved the item of interest, but in other cases, we were unable to retrieve a corresponding item in Embase, which went against the common belief that everything in MEDLINE is in Embase.

Another major obstacle was a limitation of the Ovid interface: we were unable to save a collection of articles that could easily be added to a search strategy, similar to a PubMed collection, which meant that we instead had to save searches for specific items (or sets of items, such as PMIDs connected with the Boolean OR). With thousands of articles in the reference samples, rerunning the searches to return the reference samples and testing potential search terms against them meant the searches were too large for the database to handle.

It was our experience that attempting to create search sets using our identified records (a set of a few thousand records with fewer than 100 lines in the strategy) proved much more problematic for Embase to run successfully than, for example, systematic reviews with long strategies and record sets. We are unclear as to the exact reason for this, but we were regularly ejected from the database, losing our work in the process. Ultimately, it became clear that creating a search filter to identify methodology articles relating to systematic reviews in Embase was not feasible.

## DISCUSSION

Search filters are popular tools, as they speed up search strategy design and offer some level of reassurance that relevant material will be captured. However, search filters are not necessarily suitable for all purposes. For example, when Golder and colleagues investigated the feasibility of search filters for adverse effects of non-pharmaceutical interventions, they found that development of a search filter for surgical adverse effects might be feasible, but the terms used in publications of other, non-surgical interventions would not result in a useful filter [11]. A 2013 review found that none of the published diagnostic accuracy filters available achieved a sensitivity greater than 90% and a precision of around 10%, suggesting that use of diagnostic accuracy filters should not be the only approach used in systematic reviews [12]. Further, Wilczynski and colleagues found that the natures of the nursing and physical rehabilitation disciplines were not conducive to satisfactory definitions of what articles were or were not relevant to the field, making creation of filters for these disciplines impossible [13].

Term development issues, indexing practices, and technical obstacles ultimately mean that creating a search filter to identify systematic review methodology articles in Embase is not possible at this time. As was the case with both Golder and colleagues and Wilczynski and colleagues, we were not able to identify terminology that was sufficiently unique for our topic of interest [11, 13]. For example, identifying and dealing with bias is an important methodological issue in systematic reviews, but authors may also simply report on potential biases that are present rather than discuss it from a methodological standpoint.

VOSViewer was chosen as a tool for this project because it was successful in the literature for identifying keyword frequencies [14, 15], but using the program did not help us reach our goal. As illustrated in the appendix, VOSViewer showed that the highly relevant subject headings “analytic method” and “analysis of variance” were common to only nine records. We were also surprised to see that other subject headings that we expected to be present, such as those related to other statistical methods or even the general subject heading “methodology,” did not appear.

The analysis of keywords appearing in the title and abstract fields also did not provide us with major insights into how to structure a filter. The fuzzy nature of keyword terminology for the purposes of this search filter project underscores the importance of consistent and accurate indexing, both in the development of search filters and in day-to-day bibliographic searching. Excessively liberal use of subject headings, such as tagging a systematic review with the subject heading “systematic review (topic)/,” is counterproductive when the user must rely heavily on indexing.

Other elements of Embase indexing practices are also problematic. Embase has a reputation for being “over indexed,” using many more index terms than other databases, which tends to result in large numbers of irrelevant results [16–19]. Embase also makes use of “candidate terms” in its indexing. Candidate terms are not controlled vocabulary but are suggested terms thrown in by individual indexers when no suitable Emtree term is available, which can lead to inflated results. Some candidate terms may eventually be added to Emtree, but others remain unstandardized tags [20].

Embase’s treatment of MEDLINE records further confuses the indexing issue. According to the Embase website, “Indexed MEDLINE records are delivered to Elsevier on a daily basis, and are incorporated into Embase after de-duplication with records already indexed by Elsevier” [21]. The implications here are twofold. First, items that are unique to MEDLINE are not re-indexed for Embase, even though there is a process in place to map Medical Subject Headings (MeSH) to Emtree headings. This could explain why both “clinical trial” and “clinical trials” (which could make up part of a larger phrase such as the MeSH term “Clinical Trials as Topic”) appeared in the VOSViewer analysis of subject headings. Second, Embase only imports indexed article records from MEDLINE but not non-indexed MEDLINE records. Some records remain non-indexed in MEDLINE for an extended period, so this would account for some MEDLINE records not being present in Embase.

In our practice, we have found that a search in Embase typically retrieves many more irrelevant records than an equivalent search in PubMed or MEDLINE, which prompted us to initiate this filter project. Further discussions with staff who created and maintain the PubMed subset confirmed that their team used a manual screening process to build the subset and they did not have a filter-type solution for PubMed built into their processes. In the creation of a filter or subset of this type, it seems that human intervention (i.e., screening) is needed for the correct identification of relevant articles and that database searches or algorithms are not sufficient. Given the challenges we faced with Embase coupled with confirmation that others were unable to rely on search filters to tease out items of interest to an arguably better structured database, we decided to accept our project for what it was: a failure. Compounding our inability to narrow down systematic review methodology articles in Embase, in June 2018, the PubMed subset was discontinued and is no longer available for use, leaving searchers once more without any means of narrowing search results to articles focused on systematic review methodology.

There remains a lack of filters relating to knowledge synthesis methodology papers, which is further complicated by the recent demise of the PubMed subset on this topic. The development of such a filter in Embase is not realistic at this time but may be in the future. In retrospect, it would have been prudent to test a smaller sample of records to determine the feasibility of such a project before embarking on the full-scale project. While our project did not have the intended result, we can still learn from the experience. We hope that others who have similar outcomes in their work are encouraged to share those results in the literature so that all can benefit from the lessons learned.

## SUPPLEMENTAL FILE

Appendix Tables 1–3 VOSViewer twenty most common terms
