# Supplementary material for: A failed attempt at developing a search filter for systematic review methodology articles in Ovid Embase
Source: J Med Libr Assoc. 2019 Apr 1;107(2):203–9. doi: 10.5195/jmla.2019.519 (PMC6466493; doi:10.5195/jmla.2019.519)
Supplement: Appendix [file jmla-107-203-s001.pdf]

JMLA Journal of the Medical Library Association

SC
Supplemental content to
J Med Libr Assoc. Apr;107(2):dx.doi.org/10.5195/jmla.2019.519
www.jmla.mlanet.org
© Neilson, Lê 2019

A failed attempt at developing a search filter for systematic review methodology articles in Ovid Embase

Christine Neilson, AHIP; Mê-Linh Lê, AHIP

APPENDIX

Tables 1–3 VOSViewer twenty most common terms

Table 1 The twenty most common terms located in the subject heading field, as identified by VOSViewer software

Frequency range	Term	(Count)	Term	(Count)
1–5		–		
6–10	• analysis of variance<br>• analytic method<br>• clinical practice guidelines<br>• comparative effectiveness	(9)<br>(9)<br>(10)<br>(10)	• comparative effectiveness research<br>• evidence-based medicine<br>• grade<br>• heterogeneity	(9)<br>(10)<br>(10)<br>(9)
11–15	• access to information<br>• algorithm<br>• clinical research	(13)<br>(15)<br>(13)	• decision making<br>• human	(11)<br>(13)
16–20	• accuracy<br>• adult<br>• clinical trial	(19)<br>(19)<br>(19)	• clinical trial<br>• meta-analysis	(19)<br>(17)
21–25		–		
26–30		–		
31–35	bias	(34)		
36–40		–		
41+	article	(267)		

Note: Candidate terms, as-yet unstandardized descriptors that may one day become subject headings, are indexed in the Ovid Embase Subject Heading Field.

JMLA Journal of the Medical Library Association

SC
Supplemental content to
J Med Libr Assoc. Apr;107(2):dx.doi.org/10.5195/jmla.2019.519
www.jmla.mlanet.org
© Neilson, Lê 2019

Table 2 The twenty most common terms located in the title field, as identified by VOSViewer software

Frequency range	Term	(Count)	Term	(Count)
1–50	• effect	(45)	• model	(48)
	• intervention	(43)	• outcome	(44)
51–100	• approach	(52)	• guideline	(54)
	• bias	(67)	• quality	(91)
	• clinical trial	(59)	• reporting	(64)
	• comparison	(51)	• review	(68)
	• data	(79)		
101–150	• analysis	(101)	• study	(114)
	• method	(102)	• trial	(103)
151–200	• evidence	(173)		
201–250	• meta analysis	(244)	• systematic review	(229)

Table 3 The twenty most common terms located in the abstract field, as identified by VOSViewer software

Frequency range	Term	(Count)	Term	(Count)
1–100		–		
101–200	• checklist	(174)	• heterogeneity	(175)
	• health	(170)	• synthesis	(197)
201–300	• abstract	(204)	• report	(278)
	• database	(300)	• search	(264)
	• decision	(267)	• test	(287)
	• development	(243)	• treatment effect	(238)
	• item	(224)		
301–400	• author	(394)	• process	(344)
	• estimate	(321)	• recommendation	(345)
	• journal	(400)	• reporting	(339)
401–500		–		
501–600	• model	(565)		
